# EvalTest: a web-based tool for the assessment of diagnostic test performance

**DOI:** 10.11613/BM.2026.020101

**Published:** 2026-04-15

**Authors:** Nassim Ayad

**Affiliations:** Modeling and Biostatistics Unit, Pasteur Institute of Algeria, Algiers, Algeria

**Keywords:** accuracy, diagnostic test, evaluation, laboratory medicine, biostatistics

## Abstract

**Introduction:**

Accurate diagnostic tests are essential in clinical and laboratory medicine. Evaluation of diagnostic test performance requires advanced statistical expertise and the use of specialized or proprietary software. The aim of this work is to develop an open-source R Shiny application for facilitating diagnostic test evaluation by integrating statistical rigor with user-friendly interactivity.

**Materials and methods:**

The EvalTest application was developed in the R programming language using the Shiny framework and released as an open-source package on the Comprehensive R Archive Network, with the full source code available on GitHub. In addition, the application is available on a cloud platform and accessible *via* web browser. Statistical formulas used to compute diagnostic performance indicators and their confidence intervals were implemented within the R environment to ensure transparency and reproducibility.

**Results:**

The developed application provides an interactive interface for importing Excel datasets, setting test variable type, selecting test and reference variables, and specifying disease prevalence. It automatically computes key diagnostic performance indicators, including sensitivity, specificity, predictive values, likelihood ratios, accuracy, the Youden index, and the area under the curve (AUC), along with their 95% confidence intervals. EvalTest also generates confusion matrices, receiver operating characteristic (ROC) curves with confidence bands, and identifies the optimal cutoff for quantitative tests. All numerical and graphical outputs can be exported in suitable formats to facilitate reporting and documentation.

**Conclusions:**

EvalTest provides an open and reproducible solution for diagnostic test evaluation by computing and visualizing key performance measures with their confidence intervals, supporting its use in clinical and research settings.

## Graphical abstract

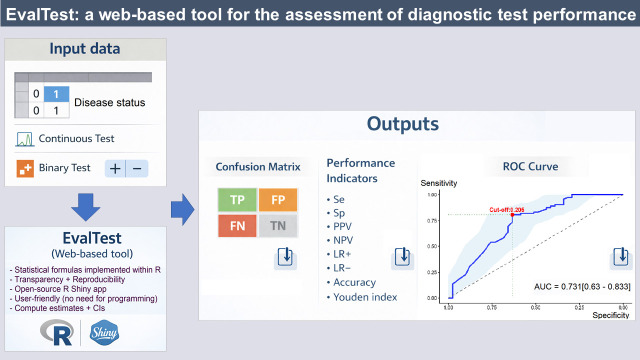

## Highlights

• EvalTest provides an open-source, web-based tool for diagnostic test evaluation

• It computes key diagnostic performance metrics and performs ROC curve analysis with transparent methods

• The application enhances reproducibility and accessibility in clinical and research diagnostics

## Introduction

A diagnostic test is a medical, clinical, or paraclinical examination designed to assist clinicians in establishing a diagnosis and guiding patient care decisions (*1*). Depending on its intended application, test performance may be evaluated by its ability to discriminate between clinical states (*e.g.* presence *versus* absence of disease) or to detect clinically meaningful changes over time, such as those defined by critical difference or reference change values. Before implementation in clinical practice or laboratory medicine, a diagnostic test requires careful performance assessment, analogous to the evaluation of a drug or therapeutic intervention, as its accuracy directly impacts diagnostic decisions, treatment choices, and patient outcomes (*2*). Such evaluations are typically performed by manufacturers, independent investigators, or clinical laboratories, depending on the context, and complement regulatory requirements, such as those defined by Regulation (EU) 2017/746 on the In-Vitro Diagnostic Regulation (IVDR) (*3*).

Performance is typically assessed by comparing the test results to those of a reference standard, defined as the best available diagnostic test for the disease of interest or, when appropriate, clinical outcomes, reasonably applicable in clinical or research settings (*1*). Common statistical indicators include sensitivity (ability to correctly identify diseased individuals), specificity (ability to correctly identify non-diseased individuals), positive and negative predictive values (probability that positive or negative test results reflect true disease status), likelihood ratios (ability of positive or negative tests results to increase or decrease, respectively, the probability of disease), accuracy (overall correct classification), and the area under the receiver operating characteristic (ROC) curve. These metrics provide critical insights into a test’s discriminative performance and support clinicians in selecting appropriate diagnostic strategies (*4*). Despite their importance, the computation and interpretation of diagnostic performance metrics often require advanced statistical expertise and reliance on specialized or proprietary software (*e.g.* MedCalc, Analyse-it, XLSTAT). Such tools may limit accessibility due to licensing costs, restricted platforms, or the need for advanced training, and often provide limited transparency regarding the underlying calculations, thereby hindering reproducibility and independent verification of results. These limitations can pose significant barriers for healthcare professionals and researchers, particularly in resource-limited settings.

The aim of the present work was therefore to develop an open-source, accessible, and flexible software solution for the evaluation of diagnostic test performance. To address the identified challenges, we developed EvalTest, an open-source, web-based application. By providing an interactive, user-friendly, and programming-free platform that integrates standard diagnostic performance metrics in a transparent and reproducible manner, EvalTest seeks to bridge the gap between established statistical methodology and its practical application in laboratory medicine and clinical research.

## Materials and methods

### Materials

In this work, a synthetic dataset was generated to illustrate the functionalities and workflow of the developed application. The generated dataset, entitled data.xlsx, contained many observations distributed across three columns: Disease, Test1, and Test2. The Disease column represented the reference variable coded as binary values (1 = disease, 0 = no disease) and was generated using a binomial distribution. Test1 corresponded to a quantitative test variable generated from chi-square (χ^2^) distribution. The chi-square distribution was chosen for Test1 to generate a positive-valued, right-skewed quantitative variable. Test2 represented a qualitative (binary) test variable generated using a binomial distribution. Assumed disease prevalence in the population was 0.1, reflecting a realistic low-prevalence scenario commonly encountered in diagnostic testing contexts. The generated dataset consisted of a total of 113 observations, including 72 diseased and 41 non-diseased individuals, according to the reference variable. This dataset was created exclusively for testing and demonstration purposes and does not represent real patient or clinical data (Supplementary Material).

### Methods

The open-source application shown here was developed within RStudio environment (version 2025.05.0) with R programming language (version 4.5.1) which are available to download for free at https://posit.co/downloads/ and https://cran.r-project.org/, respectively (*5*, *6*). The application was built using the shiny package and relies on several established R packages to support data import, statistical analysis, visualization and outputs export (*7*-*15*). Specifically, the binom package (version 1.1) was used to compute Wilson confidence intervals for proportion-based metrics, while the pROC package (version 1.19.0.1) was employed for ROC analysis (*8*, *13*). All packages’ versions are reported in the reference list to ensure full reproducibility. EvalTest (version 1.0.5) has been developed as an R package and officially released and publicly available through the Comprehensive R Archive Network (*16*). This means that the application can be installed as an R package and run from a local instance of R on a local machine running a Linux, Windows, or macOS-based operating system. It is worth noting that the performance will depend on the hardware on which you are running R and RStudio. In addition, EvalTest is available on the Shiny.io cloud platform and accessible *via* web browser at https://nassimayad.shinyapps.io/EvalTest/, without requiring local installation. The full codebase for the application can be found at https://github.com/NassimAyad87/EvalTest. The version used to generate the figures and results presented in this manuscript corresponds to release v1.0.5 of the repository (commit ec6306d). The application’s modularity enables interested users to fork this repository and easily develop and add new modules as they wish.

### Statistical analysis

The evaluation of diagnostic tests relies on four fundamental outcomes obtained by comparing test results with the gold standard. The true positives (TP) are the number of diseased individuals which correctly identified as positives, while the true negatives (TN) correspond to the number of non-diseased individuals correctly identified as negatives. Conversely, the false positives (FP) refer to the number of non-diseased individuals incorrectly identified as positives, and the false negatives (FN) indicates the number of diseased individuals incorrectly identified as negatives (*17*). The open-source application implements these definitions to quantify TP, TN, FP, and FN and present them in a confusion matrix table. These outcomes form the basis for calculating statistical performance indicators such as sensitivity and specificity.

Statistical indicators implemented in the application are mathematically defined in this chapter. These measures evaluate the ability of a test to discriminate between diseased and non-diseased individuals. All estimates are reported with their corresponding confidence intervals.

Test sensitivity (Se) is the probability that a diseased individual tests positive, whereas test specificity (Sp) is the probability that a non-diseased individual tests negative (*17*). The formulas used in their computations are shown in equations (Eq.) 1 and 2. The accuracy of a test is its ability to differentiate the patient and healthy cases correctly. To estimate the accuracy of a test, we should calculate the proportion of true positive and true negative in all evaluated cases, as shown in Eq. 3 (*18*).



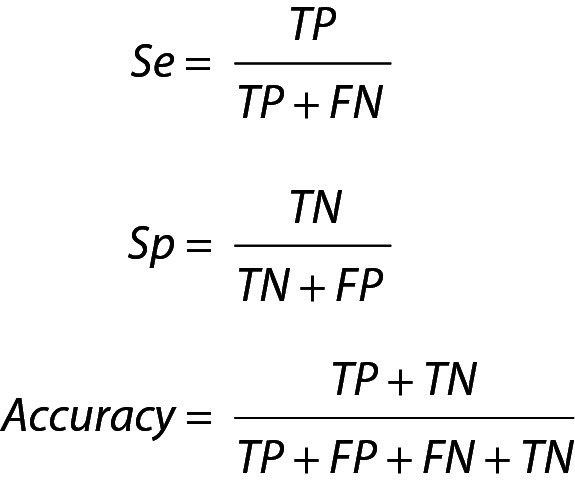



For proportions such as sensitivity, specificity, and accuracy, confidence intervals (CIs) were calculated using the Wilson binomial confidence interval implemented in the binom package (*8*). Wilson interval is recommended because it performs better than the traditional Wald interval, especially with small samples or proportions close to 0 or 1 (*19*). Given the observed proportion (p), its denominator (n), and the quantile of the standard normal distribution for the desired confidence level (z), the Wilson interval CI_Wilson_ is calculated as shown in Eq. 4.



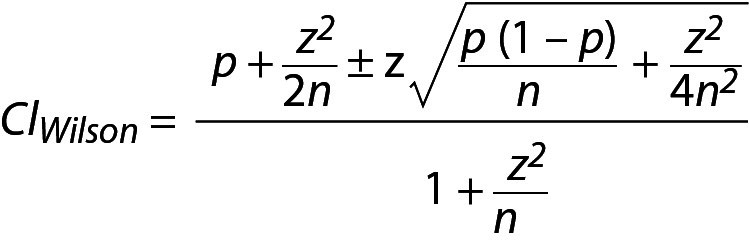



The positive predictive value (PPV) is the probability that a person has a disease if his test is positive. Similarly, we can define the negative predictive value (NPV), the probability that a person does not have a certain disease when the test is negative. PPV and NPV are determined by both the sensitivity and specificity of the test, as well as the prior probability of disease. When no other information is available, this probability is represented by the population disease prevalence (pr) specified by the user in the application (*17*). The sample prevalence is not used, as it does not provide additional information when a population-level prevalence is known. PPV and NPV are therefore derived from Bayes’ theorem (Eq. 5 and 6) using the estimated sensitivity and specificity together with the user-defined prevalence parameter, rather than the observed sample prevalence. Their confidence intervals (CI_PPV_, CI_NPV_) were derived by propagation of uncertainty using the confidence bounds of sensitivity and specificity. Since PPV and NPV are functions of Se and Sp, their uncertainty depends on the variability of Se and Sp. The lower and upper bounds of PPV and NPV were determined by evaluating all combinations of Se and Sp confidence interval limits and taking the minimum and maximum resulting values, respectively (Eq. 7 and 8). This approach provides a conservative, distribution-free estimate of uncertainty and is particularly appropriate in small samples. Analytic variance estimation of predictive values relies on asymptotic normality, which may perform poorly in small samples, whereas bootstrap-based confidence intervals may be unstable under limited resampling variability and can require substantial computational effort when a large number of resamples is required (*20*).



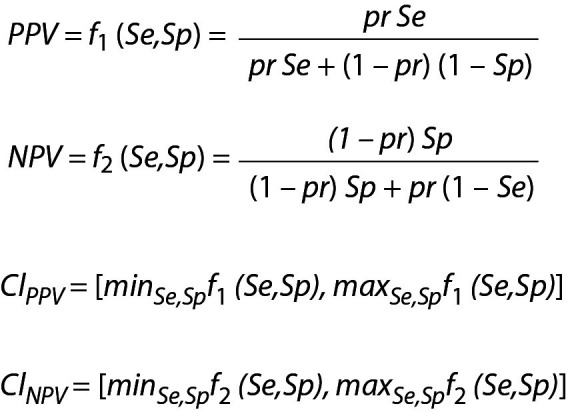



The likelihood ratios (LR^+^ and LR^-^) summarize how many times more (or less) likely patients with the disease are to have that particular test result than patients without the disease. More formally, it is the ratio of the probability of the specific test result in people who do have the disease to the probability in people who do not (*21*). Their calculation formulas are shown in Eq. 9 and 10. Their confidence intervals (CI_LR+_ and CI_LR-_) were estimated in logarithm scale, following the approach described by Simel *et al.* (*22*). They are computed as shown in Eq. 11 and 12.



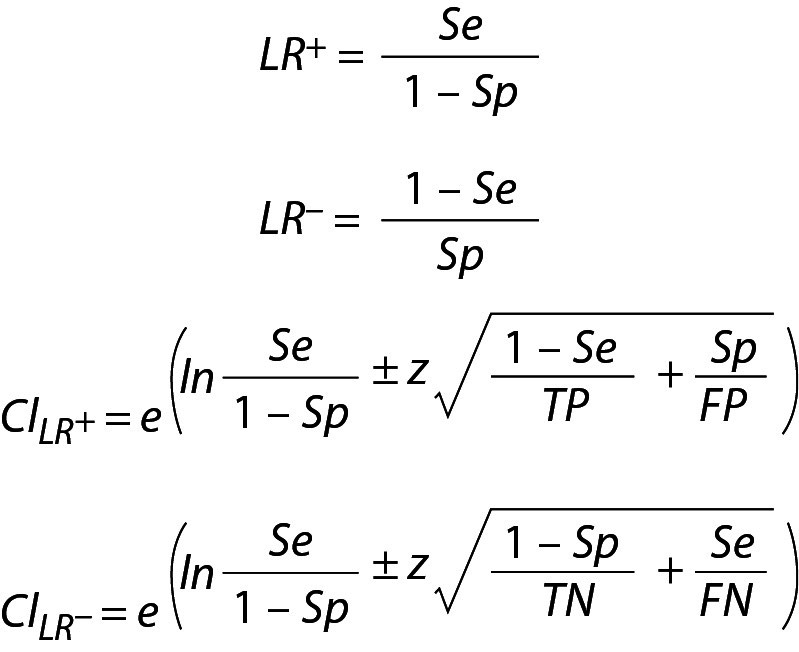



The Youden index (J) is a global measure of a diagnostic test’s discriminative ability, ranging from 0 to 1, where 0 indicates no discriminative power (random classification) and 1 indicates a perfect diagnostic test (*23*). It is calculated as shown in Eq. 13. Under the common assumption that sensitivity, estimated from diseased subjects, and specificity, estimated from non-diseased subjects, represent independent proportions, the variance of the Youden index can be approximated by the sum of the binomial variances of sensitivity and specificity. Subsequently, confidence interval of Youden index (CI_J_) is computed as shown in Eq. 14.



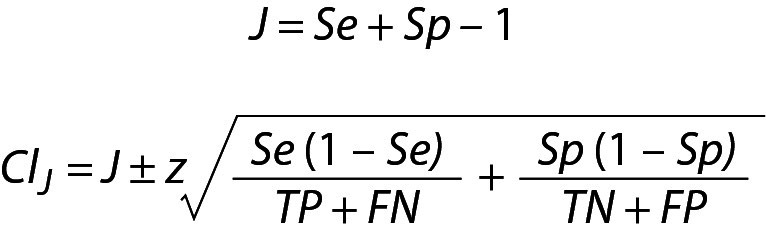



The receiver operating characteristic (ROC) curve was generated using the framework implemented in the pROC package and the area under the curve (AUC) was calculated accordingly (*13*, *24*). The optimal threshold of the continuous test variable was defined as the value of (t) that minimizes the Euclidian distance d(t) between the ROC curve point (1 − Sp, Se) and the top-left corner of the ROC space, as expressed in Eq. 15 (*25*). In this formulation, Se(t) and Sp(t) denote the sensitivity and specificity at a given cutoff t, respectively. Using this threshold, the continuous test variable was dichotomized to classify each observation in the example dataset as test-positive or test-negative. These binary classifications were then used to construct the corresponding confusion matrix, from which sensitivity, specificity, predictive values, likelihood ratios, and other performance metrics were calculated. This stepwise procedure illustrates how the ROC analysis directly informs the computation of diagnostic performance measures for the dataset. Confidence intervals for sensitivity estimates along the ROC curve were obtained using nonparametric bootstrap resampling with 5000 iterations, as implemented in the pROC package (*13*). These interval estimates were used solely to construct the graphical confidence band around the ROC curve. As bootstrap procedures rely on random resampling and the application is interactive, a fixed random seed was not imposed; therefore, minor graphical variation of the confidence band may occur across runs. The confidence interval for the AUC was calculated using DeLong’s nonparametric method (*24*). Additional graphical enhancements, including the display of the optimal cut-off point and AUC value with its confidence interval, were implemented using the ggplot2 package (*10*).



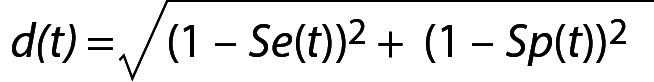



To ensure computational robustness, all formulas were implemented with error-handling conditions. Whenever the denominator of a proportion was equal to zero, the function returned a missing (NA) value to prevent the application from freezing or encountering errors.

All confidence intervals were calculated at the 95% confidence level, using a standard normal quantile of z = 1.96. All estimates and confidence intervals were rounded to three decimal places.

## Results

### Application overview

**Figure 1:**
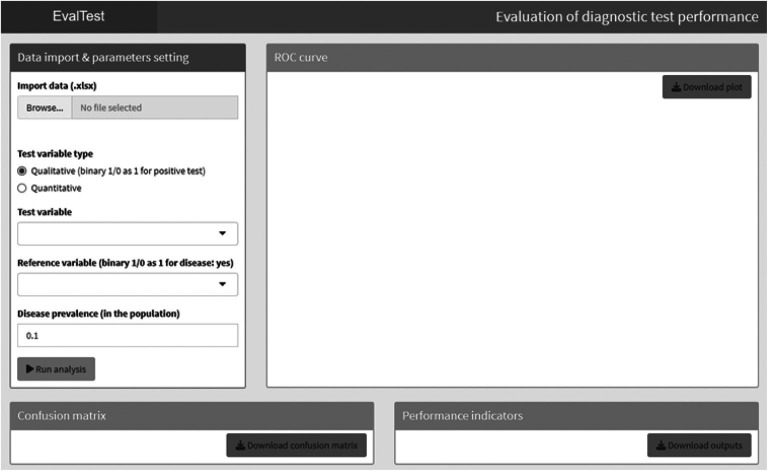
EvalTest user interface layout.

The EvalTest application provides a user-friendly graphical interface for evaluating diagnostic test performance through an interactive R Shiny environment. As shown in Figure 1, the interface is organized into clearly defined sections that guide users through the analysis workflow.

On the left panel, users can import their dataset in Excel format (.xlsx), specify the test variable type (qualitative or quantitative), select the corresponding columns for the test and reference (disease status) variables, and input the disease prevalence within the study population. Disease prevalence value can be adjusted manually by entering a number or using the up and down arrow controls. The Run analysis button initiates the computation process. The central and lower panels display the main analytical outputs, including the ROC curve, the confusion matrix (contingency table), and the diagnostic performance indicators. Each component is accompanied by a download option, allowing users to export the ROC plot, confusion matrix, and calculated metrics in appropriate formats.

This intuitive layout ensures a seamless workflow from data upload to results interpretation, making EvalTest accessible to healthcare professionals and researchers with or without advanced statistical or programming expertise.

### Data input and preprocessing

The example dataset consisted of 113 observations and included one binary reference variable indicating disease status and two test variables. The reference variable defined two outcome groups, with 72 observations as disease-present and 41 as disease-absent. Test1 was a continuous variable, whereas Test2 was a binary test variable.

**Figure 2:**
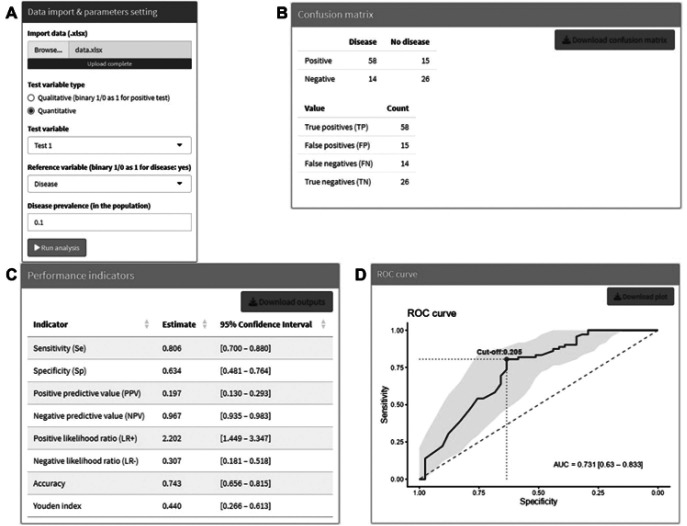
EvalTest parameters setting and outputs. (A) Data import and parameters setting panel showing uploaded data, variable selection, and parameter configuration. (B) Confusion matrix generated by EvalTest, illustrating the classification results for the selected quantitative test variable Test1 and the reference variable Disease, and displaying the true positives (TP = 58), false positives (FP = 15), false negatives (FN = 14), and true negatives (TN = 26). (C) Diagnostic performance indicators computed by EvalTest, derived from the analyzed dataset data. (D) ROC curve generated by EvalTest for the quantitative test variable Test1, showing the optimal cut-off point and the area under the curve (AUC) with its 95% CI. The x-axis represents specificity displayed in reversed direction (*i.e.* equivalent to 1-specificity). The shaded area corresponds to the 95% bootstrap confidence band.

In the “Data import & parameters setting” panel, users can upload their dataset in Excel format (.xlsx) by clicking the ‘Browse’ button (Figure 1). Before uploading, the dataset should contain a minimum of two distinct columns: one for the test variable (either qualitative binary coded as 1/0 or quantitative) and another for the reference variable, representing the disease status (binary: 1 = diseased, 0 = non-diseased). The selected columns must not contain missing values to ensure valid computation. Once the dataset is successfully uploaded, a blue ribbon appears, indicating “Upload complete”. Then, users specify the type of test variable (Qualitative or Quantitative) and select the corresponding columns for both the test and reference variables from the drop-down menus. The disease prevalence in the study population can then be entered manually or adjusted incrementally using the up and down arrow controls within the prevalence input field. For the example analysis presented in this work, Test1 was selected as the quantitative test variable and Disease as the reference variable, with the disease prevalence parameter set to 0.1 (Figure 2A).

To enhance data reliability, EvalTest automatically performs input validation and displays error messages when inconsistencies are detected. For instance, if the user selects “Qualitative” as the test variable type but uploads continuous data, an error appears indicating: “For qualitative tests, the test variable must contain only 0 and 1 values.”. Similarly, if the reference variable includes invalid entries, the application displays: “The reference variable must contain only 0 and 1 values.”. In the case of quantitative analyses, if the selected test variable is not numeric, the system halts the process and notifies the user with the message: “For quantitative tests, the test variable must be numeric.” Furthermore, after removing missing values, if the reference variable no longer includes both outcome levels (0 and 1), an additional alert is triggered stating: “The reference variable must contain both 0 and 1 after removing missing values.” These built-in checks help prevent input errors and guide users toward proper dataset formatting. This preprocessing step ensures that the uploaded dataset is correctly structured and ready for subsequent diagnostic performance analysis within the EvalTest application.

### Confusion matrix

Upon activation of the “Run analysis” button (Figure 2A), EvalTest generated a detailed confusion matrix summarizing the classification of individuals according to the test and reference results. The matrix displays the counts of true positives (TP = 58), false positives (FP = 15), false negatives (FN = 14), and true negatives (TN = 26), which are automatically computed from the imported dataset and presented both in matrix form and as a summarized list (Figure 2B). For continuous test variables, such as Test1 in this example, the application first determined the optimal threshold from the ROC analysis and then dichotomized the test results into positive and negative outcomes based on this cutoff. The confusion matrix could also be downloaded in Excel format for further documentation or reporting (Figure 2B).

### Diagnostic performance indicators

Based on the confusion matrix counts, EvalTest automatically computes key diagnostic performance indicators, including sensitivity, specificity, predictive values, accuracy, likelihood ratios, and the Youden index as shown in Figure 2C. Each metric is displayed together with its 95% confidence interval (CI). The availability of the underlying confusion matrix ensures transparency by allowing readers to identify the numerators and denominators used for each performance estimate. All computed results are presented in a tabular format and can be exported as an Excel file for further use.

### ROC curve analysis and cutoff determination

EvalTest displayed the ROC curve, illustrating the trade-off between sensitivity and specificity across all possible thresholds. The application automatically identified and highlighted the optimal cutoff point of the test variable, annotating it on the ROC plot with its corresponding sensitivity and specificity values. In the example shown, the optimal cutoff was approximately 0.205. The AUC was 0.731 (95% CI: 0.630–0.833). The ROC plot displayed the confidence band around the curve, illustrating the uncertainty associated with the estimated ROC curve. Users can export the plot in publication-ready quality for inclusion in reports or manuscripts (Figure 2D).

## Discussion

In the illustrative example, the selected cut-off resulted in high sensitivity and moderate specificity. The imbalance between sensitivity and specificity is consistent with a cut-off chosen to favor case detection. The positive predictive value was low and the negative predictive value was high, illustrating the strong influence of the low disease prevalence in the population on post-test probabilities. Bayes formulas were used to estimate predictive values to obtain more conservative and generalizable results when the sample prevalence is not representative of the target population. The likelihood ratios indicate that a positive test result produces only a modest increase in disease probability, whereas a negative test result substantially reduces the likelihood of disease. Overall performance measures, including accuracy and the Youden index, summarize moderate discriminatory effectiveness at the chosen threshold. The AUC indicates moderate discriminative ability of the continuous test. The uncertainty associated with these estimates should be carefully considered.

EvalTest provides a user-friendly and open-source tool for evaluating diagnostic test performance, integrating advanced statistical methods within an accessible web-based interface. Unlike commercial tools such as MedCalc, Analyse-it, or XLSTAT, EvalTest requires no programming skills, proprietary licenses, or software installation, facilitating broader accessibility and reproducibility. The application automatically computes key diagnostic indicators such as sensitivity, specificity, predictive values, likelihood ratios, accuracy, and the Youden index, along with 95% CI and ROC curve visualization. Built-in data validation, error handling and transparent reporting of underlying counts enhance robustness, clarity, and allow users to verify results. The application’s ability to process both binary and continuous test variables, automatically determine optimal thresholds, and generate publication-ready outputs constitutes a major practical advantage over many existing tools.

Several limitations should be acknowledged, EvalTest currently supports only single-test analyses. It does not include modules for comparative ROC analysis, multivariable adjustment, or time-dependent ROC curves. Additionally, confidence interval estimates - particularly for likelihood ratios and predictive values - may exhibit increased uncertainty in small sample sizes or in settings with extreme or zero cell counts. In such cases, the application returns NA for the affected estimates, ensuring that undefined or misleading values are not displayed. In addition, predictive values depend directly on user-specified prevalence assumptions; while this allows clinically relevant post-test probability estimation when samples are not representative, inappropriate prevalence inputs may affect interpretation. Future developments will focus on incorporating comparative and multivariate analyses, addressing uncertainty arising from user-specified prevalence, implementing continuity corrections or alternative estimation methods and extending compatibility with additional data formats.

EvalTest bridges the gap between statistical methodology and clinical application, offering an open, transparent, and reproducible tool for diagnostic test evaluation. Its accessibility and methodological rigor make it a valuable addition to the resources available for clinicians, laboratory scientists, and researchers.

## Data Availability

All data generated and analyzed in the presented study are included in this published article and its supplementary files.
